# Enhanced Attentional Network by Short-Term Intensive Meditation

**DOI:** 10.3389/fpsyg.2019.03073

**Published:** 2020-02-07

**Authors:** Seoyeon Kwak, So-Yeon Kim, Dahye Bae, Wu-Jeong Hwang, Kang Ik Kevin Cho, Kyung-Ok Lim, Hye-Yoon Park, Tae Young Lee, Jun Soo Kwon

**Affiliations:** ^1^Department of Brain and Cognitive Sciences, College of Natural Sciences, Seoul National University, Seoul, South Korea; ^2^Department of Psychology, Duksung Women’s University, Seoul, South Korea; ^3^Institute of Human Behavioral Medicine, Medical Research Center, Seoul National University, Seoul, South Korea; ^4^Department of Psychiatry, National Institute of Forensic Psychiatry, Ministry of Justice, Gongju-si, South Korea; ^5^Department of Psychiatry, College of Medicine, Seoul National University, Seoul, South Korea

**Keywords:** meditation, mindfulness training, Templestay, attention, attention network test

## Abstract

While recent studies have suggested behavioral effects of short-term meditation on the executive attentional functions, functional changes in the neural correlates of attentional networks after short-term meditation have been unspecified. Here, we conducted a randomized control trial to investigate the effects of a 4-day intensive meditation on the neural correlates of three attentional functions: alerting, orienting, and executive attention. Twenty-three participants in meditation practice and 14 participants in a relaxation retreat group performed attention network test (ANT) during functional magnetic resonance imaging both before and immediately after intervention. The meditation group showed significantly improved behavioral performance in the executive control network in ANT after the intervention. Moreover, neural activities in the executive control network, namely, the anterior cingulate cortex (ACC) and dorsolateral prefrontal cortex (DLPFC), were also significantly increased during the ANT after meditation. Interestingly, neural activity in the right ACC was significantly predicted by behavioral conflict levels in each individual in the meditation group, indicating significant effects of the program on the executive control network. Moreover, brain regions associated with the alerting and orienting networks also showed enhanced activity during the ANT after the meditation. Our study provides novel evidence on the enhancement of the attentional networks at the neural level via short-term meditation. We also suggest that short-term meditation may be beneficial to individuals at high risk of cognitive deficits by improving neural mechanisms of attention.

## Introduction

Meditation is a form of mental training that originated from Buddhist traditions and that aims to enhance an individual’s core psychological capacities. Because of its positive effects on physical and psychological conditions, meditation has received much attention in psychological and clinical research in the past several decades (Ludwig and Kabat-Zinn, 2008; Rubia et al., 2009; van der Velden and Roepstorff, 2015). A review summarized the effects of meditation practice on self-regulation in terms of the following four components: attention regulation, body awareness, emotion regulation, and perspective on the self (Holzel et al., 2011). Beyond these four components, meditation traditions emphasize the importance of attention in the practice. Indeed, attention is known to actively interact with the components of meditation: increasing one’s ability to control attention (attention regulation), maintaining focus on the body (body awareness), and bringing non-judgmental awareness toward negative information (emotion regulation). Therefore, many behavioral studies reported the enhancement of executive attention in meditation practitioners (Anderson et al., 2007; Chan and Woollacott, 2007; Jha et al., 2007).

In line with behavioral findings, neuroimaging studies have also found effects of meditation in brain areas related to executive attention and attentional control. One of the most consistently reported brain regions for the effects is the anterior cingulate cortex (ACC), which contributes to executive attentional functions by detecting a cognitive conflict and alerting top-down-regulated systems, such as the dorsolateral prefrontal cortex (DLPFC), to resolve the conflict (van Veen and Carter, 2002; Sridharan et al., 2008). Many previous functional MRI (fMRI) studies found reduced activation in the ACC in expert meditators compared to the control group, and a recent meta-analysis confirmed the findings (Brefczynski-Lewis et al., 2007; Holzel et al., 2007; Lutz et al., 2008; van den Hurk et al., 2010; Kozasa et al., 2012; Falcone and Jerram, 2018).

However, those previous cross-sectional studies with meditation experts were unable to exclude possible pre-existing group characteristics or the effect of accumulated meditation group within the group. To overcome these limitations, some studies have adopted short-term meditation for people with no prior experience with meditation (Tang et al., 2007; MacLean et al., 2010; Ainsworth et al., 2013). Even though these studies suggest enhanced executive attention after short-term meditation at the behavioral level, only few studies explore whether short-term meditation training can alter neural functions in regions related to executive attention. Interestingly, neuroimaging studies with the short-term meditation training presented different neural patterns compared to those with meditation experts. Instead of the decreased brain activation, which was shown in meditation experts, beginners demonstrated the increased brain activation, especially in ACC and DLPFC regions (Xue et al., 2011; Allen et al., 2012; Tomasino and Fabbro, 2016). Based on these results, previous review studies suggested increased brain activation in neural correlates of executive attention in the early stage of meditation. Thus, it is critical to discover whether short-term meditation can change neural correlates of attention and executive function online during cognitive tasks involving cognitive conflicts.

To elucidate the effects of short-term meditation, in the current study, we adapted Templestay training from the Korean Buddhist temple. The training program involves 4 days of an intensive residential meditation retreat in a Korean Buddhist temple, including combinations of different forms of meditation with a broader general mindfulness lifestyle based on Buddhist teachings. To minimize the effects of non-meditative factors, such as diet and environment, the intervention program for the relaxation group included the same diet and residence without any meditation-related activities. Our previous study suggested that the short-term intensive meditation via Templestay enhanced mindfulness and resilience after the program, and the improvements were maintained after 3 months of follow-up (Hwang et al., 2017).

In the present study, we examined the behavioral and neural functional changes of the short-term meditation program on attentional control in healthy naïve adults with no meditation experience using the attention network task (ANT) (Fan et al., 2005). Using the ANT, we tested the effects of intensive meditation during Templestay on various attention functions, namely, alerting, orienting, and executive attention, at both the behavioral and neural levels. Specifically, we hypothesized that the 4-day intensive meditation retreat would improve behavioral performance of executive attention, as previously reported (Tang et al., 2007). Further, we also expected that the short-term meditation retreat would increase activation in brain areas related to conflict resolution and executive attention during the ANT, such as the rostral and dorsal ACC and the DLPFC, as our participants were beginner meditators without prior experience (Xue et al., 2011; Allen et al., 2012; Tomasino and Fabbro, 2016). Lastly, we expected that our short-term yet intensive meditation training may also increase neural functions of alerting and/or orienting in the fronto-parietal attention network.

## Materials and Methods

### Four-Day Intensive Meditation (Templestay) Project

The Templestay project is aimed to demonstrate the effect of three nights and 4 days of intensive meditation (Templestay program) at the behavioral and neural levels. The data used in the present study are part of the Templestay Project. Participants were then randomly assigned to the meditation group or the relaxation group using a computerized algorithm based on mixed-block randomization (44 participants in the meditation group and 23 participants in the relaxation group). The two groups showed no significant demographic differences (all *p* values > 0.05). The brain imaging data were collected at two different time points: 2–3 days before and after the intervention. The recruitment procedure and intervention program are introduced thoroughly in a previous report (Hwang et al., 2017).

### Participants

Sixty-seven healthy adults with full-time employment were recruited via an Internet advertisement. Participants had no pre-existing medical, neurological, or psychiatric disorders, contraindications for MR scanning, or previous experience with meditation training. Participants who met the above qualifications were invited to visit Seoul National University Hospital for an in-person screening interview, including assessments of handedness, employment background, medical history, and health behaviors. To minimize possible placebo effects, all participants were informed that Templestay programs consisted of two types prior to participation: a Buddhism meditation type and a relaxation type. Those who were willing to be assigned to either type were enrolled in the current study. All participants provided written informed consent for participation in accordance with the Declaration of Helsinki, and the protocol was approved by the Institutional Review Board of Seoul National University Hospital Committee.

### Meditation and Relaxation Programs

Participants in the meditation group attended a retreat consisting of 4 days of intensive meditation (i.e., a modified version of a standardized Templestay program in the Buddhist temple). A structured residential retreat format was included as a part of the Templestay program to increase experimental control and compliance with training. Briefly, the program was taught by a monk who stayed in the temple, and it consisted of a total of 19 h of mindfulness training through body awareness exercises, sitting, walking and bowing meditations, mindful eating, and mindful movement. On the other hand, participants in the relaxation type spent their time in the temple as a vacation, relaxing freely around the temple, but they were restricted from being exposed to any meditation-related activities. In addition, they were required to keep a diary with the daily schedule and reflection of the day. Their activities were monitored by a trained research assistant. The intervention program for the relaxation group was designed to minimize the effects of confounding factors that are not specific to mindfulness, such as participants’ expectations, group support, environmental influences, diet, and physical activity, by providing equivalent experiences for the two groups. The program involves the same diet, group size per session, and environment in the 4-day retreat format as in the meditation group. Each subject participated in one session (three nights and 4 days), and each session included four to nine subjects. A total of 12 sessions were conducted between July 2014 and July 2015 (seven meditation and five relaxation sessions), and none of the sessions occurred concurrently. More detailed information on the program were introduced in the previous studies and Supplementary Figure 1 (Hwang et al., 2017; Kwak et al., 2019).

### Attention Network Test (ANT)

**FIGURE 1:**
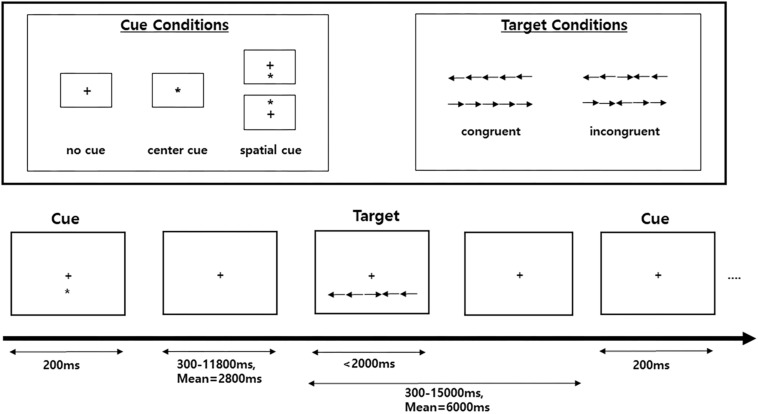
Schematic of the attention network test (ANT). A fixation cross is shown at the center of the screen, and a cue (none, center, or spatial cue) is presented for 200 ms. After 300–11,800 ms, the target (center arrow) and flankers in either the congruent condition or the incongruent condition are presented. A participant must respond to indicate whether the target is a left- or right-facing arrow with a button press.

Attentional functions were assessed with an ANT (Fan et al., 2002, 2005). This test is a combination of a flanker task and a temporal and spatial cueing task (Eriksen and Eriksen, 1974; Posner, 1980). In this study, we used three cue conditions (no cue, center cue, and spatial cue) for the cueing task and employed two target conditions (congruent and incongruent) for the flanker task (Figure 1). Specifically, the ANT requires participants to respond to whether a central arrow surrounded by a flanker points toward the right or left. The flankers were directed either in the same direction as a central arrow (i.e., congruent condition) or in the opposite direction from the target (i.e., incongruent condition). The target array, consisting of one central target and four flankers, was presented either above or below the central fixation point in each trial, and there were three cue conditions: no cue, central cue, and spatial cue. In the no-cue condition, no cue appeared before the target array, while a central cue provided temporal information that the target array would be forthcoming, and a spatial cue provided spatial information about the location of the target array with 100% validity.

In this test, the improvement of the three attention networks (alerting, orienting, and executive control networks) is assessed by measuring how participants’ reaction times (RTs) are influenced by each cue and flanker condition. The function of the alerting network was estimated by subtracting the mean RTs of the center cue condition from the mean RTs of the no-cue condition. The function of the orienting network was calculated by subtracting the mean RTs of the spatial cue conditions from the mean RTs of the center cue. The function of the executive control network (conflict effect) was evaluated by subtracting the mean RTs of congruent flanker conditions from the mean RTs of incongruent flanker conditions.

To examine the effects of the intensive meditation retreat (i.e., Templestay) on behavior and on neural functions of attention networks, the ANT was performed in an MRI scanner before and after the meditation/relaxation retreats. To check whether the participants fully understood the test, each participant underwent two practice tests (20 trials) prior to the actual task. Each participant received 210 trials during the assessment session (a total of six runs per session, 35 trials per run), and each run took 5 min and 34 s.

### Imaging Methods and Analysis

#### Brain Image Acquisition

All participants underwent MRI scanning 1–3 days before and after the retreat. Both anatomical and functional data were acquired in the sagittal plane using a 3T scanner (MAGNETOM Trio Tim Syngo MR B17, 12 channel head coil). T1-weighted images utilized 3D magnetization-prepared rapid-acquisition gradient echo (MPRAGE) with the following parameters: TR = 1670 ms, TE = 1.89 ms, FOV = 250 mm, FA = 9°, 1 mm thickness, and 208 slices. After acquiring anatomical data, fMRI images were acquired in the six functional sessions (TR = 2000 ms, TE = 30 ms, 64 × 64 matrix, FOV = 220 mm, FA = 80°, 3.4 mm thickness, no gap, and 34 axial slices).

#### Image Processing and Statistical Analyses

Image preprocessing and statistical analysis were conducted using SPM8 (Statistical Parametric Mapping, Wellcome Department of Imaging Neuroscience, Institute of Neurology, London, United Kingdom) run within MATLAB (MathWorks Inc., Natick, MA, United States). For preprocessing, data were slice-time-corrected for acquisition order (referenced to the first slice), realigned, and unwarped to correct for motion across runs. Next, the images were re-sampled with an isometric voxel size of 2 × 2 × 2 mm and spatially normalized (with trilinear interpolation and preserving the intensities of the original images) to the SPM EPI template corresponding to the MNI (Montreal Neurological Institute)-standardized brain space, followed by spatial smoothing with a Gaussian kernel of 6-mm FWHM. Motion estimates were included as covariates in the preprocessing steps, and only participants who moved less than 3 mm in the *x*, *y*, and *z* planes were included in the analyses. The time series were high-pass filtered at 128 s.

According to previous studies on the aging effect on neuroimaging, the current study included cognitive and brain data from young participants aged 40 and younger (*N* = 35 and *N* = 19 for the meditation and relaxation groups, respectively) (Ferreira and Busatto, 2013). Among the 35 participants in the meditation group, data from 12 participants were excluded from further analyses due to excessive motion (> 3 mm for *x*, *y*, or *z* planes, *N* = 8), distortion of brain data during acquisition (*N* = 1), or poor performance on our cognitive task (predetermined outliers: below 3 standard deviations from the mean accuracy, *N* = 3). In the relaxation group, data from six participants were excluded from further analyses due to excessive motion (>3 mm for *x*, *y*, and *z* planes, *N* = 4), or problems in brain data acquisition (*N* = 1). As a result, data from 23 people in the meditation group and from 14 people in the relaxation group were included in the further analyses.

Statistical analyses were performed in SPM8 using a general linear model for event-related designs. Individual events were modeled as a canonical hemodynamic response, and a whole-brain voxel-wise analysis was conducted for each participant. Using a fixed-effects analysis, each event type (three cuing events and two flanker events) was first modeled for each participant. Then, the resulting least-squares parameter estimates of the height of the modeled hemodynamic response for each condition were entered into random-effects analyses. For all random-effects analyses, we used combined intensity and cluster size thresholds of *p* < 0.005 with a minimum cluster size of 20 contiguous voxels (*k* ≥ 20) to achieve a reasonable balance between type I and type II errors. Lieberman and Cunningham (2009) have suggested that voxel-wise alpha levels of *p* < 0.005 and *k* > 20 resulted in a good trade-off of type I and type II statistical errors in fMRI research, and this threshold has been approved in previous fMRI studies (Lieberman and Cunningham, 2009; Kim and Giovanello, 2011; Gotts et al., 2012; Hur et al., 2016; Kim et al., 2018). For significant interaction effects found in the whole-brain analyses, we conducted *post hoc* analyses using beta coefficient values of the activated brain regions from each participant in each group in each condition. Voxels of interest (VOIs) were defined by 4-mm-radius spheres around the maxima of the clusters found in the significant group contrast map, and then beta values of each VOI were extracted for each. Finally, multiple regression analyses were conducted using behavioral scores as a factor and brain activity levels as a dependent variable.

#### ANT Analysis

The behavioral data from the ANT were analyzed with three-way mixed ANOVA with the timing (pre, post) and task condition as within-subject factors and the group (meditation vs. relaxation) as a between-subjects factor, using each of the attention network scores as dependent variables. *Post hoc* analyses were performed using paired *t* tests to test how each condition in the ANT was changed by time point. A cognitive subtraction approach (described in the section “Materials and Methods”) was used to assess the improvement of each attentional network.

## Results

### Demographic Characteristics

The demographic statistics for each group are summarized in Table 1. There were no significant differences between the groups.

**TABLE 1:** Demographic and clinical characteristics.

**Variables**	**Meditation (*N* = 23)**	**Relaxation (*N* = 14)**	**Statistical differences**	
			**χ^2^, *F*, or *T***	***p*-value**
Sex (male/female)	4/19	5/9	1.59	0.25
Handedness (right/left)^†^	19/4	14/0	2.73	0.28
Age (years, ±SD)	30.09 ± 4.60	31.43 ± 5.50	−0.80	0.43
Education (years, ±SD)	16.70 ± 1.78	17.61 ± 1.67	−1.55	0.13
Religion (%)			0.34	0.57
None	17 (73.9%)	10 (71.4%)		
Buddhism	4 (17.4%)	1 (7.1%)		
Catholic	1 (4.3%)	2 (14.3%)		
Presbyterian	1 (4.3%)	1 (7.1%)		
Socioeconomic Status (mean, ±SD)^‡^				
Participants’	2.57 ± 0.59	2.64 ± 0.63	−0.38	0.71
Participants’ parents	2.52 ± 0.79	2.86 ± 0.95	−1.16	0.25

*^†^Annett Handedness scores; ^‡^Hollingshead socioeconomic status index, where 1–2 = high and 4–5 = low.*

### Behavioral Performance

**FIGURE 2:**
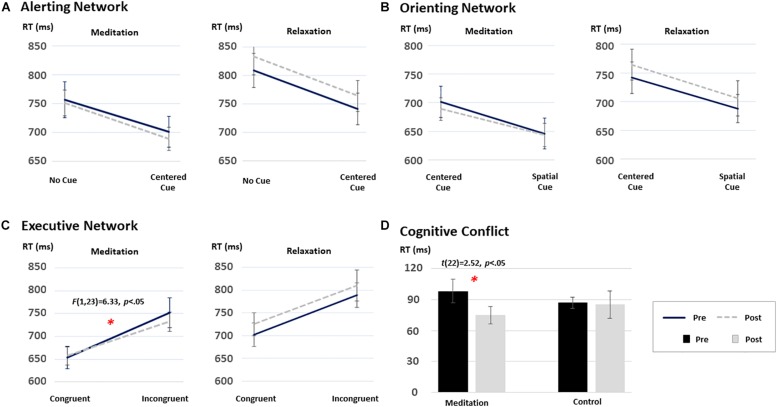
The time and group interaction effect on the performance on the ANT: **(A)** the alerting network, **(B)** the orienting network, **(C)** the executive network, and **(D)** cognitive conflict effect. Among the three attentional networks, only the executive network demonstrated significant improvement after the short-term meditation [*F*(1,35) = 2.29, *p* = 0.13]. ^∗^*p* < 0.05.

To test the effects of intensive meditation on the improvement of attention networks, 2 [Time (pre/post), within-subject factor] × 2 (Task condition for each network, within-subject factor) × 2 (Group, between-subjects factor) mixed ANOVAs were conducted for RT data in each attention network. Figure 2 shows the results from each test. Before participating in the program, no differences between the two groups were found for alerting, orienting, and executive control networks (all *p*s > 0.05). Although a three-way interaction was not significant [*F*(1,35) = 2.29, *p* = 0.13], performance differences were revealed for the executive control network after participation in the meditation program.

Specifically, a 2 (time) × 2 (task condition) repeated-measures ANOVA on the RT data from each group revealed distinctive patterns of results. That is, a significant Time × Congruency interaction was found only in the meditation group [*F*(1,22) = 6.33, *p* < 0.05], although a main effect of congruency was significant in both groups [*F*(1,22) = 94.29, *p* < 0.05 and *F*(1,13) = 95.03, *p* < 0.05, for the meditation and the relaxation groups, respectively]. To further test the significant interaction effect in the meditation group, *post hoc* analyses were carried out using paired *t*-statistics on RTs in each congruent condition at each time point (i.e., pre/post). The test revealed significant changes in the executive control network in the meditation group after the intervention compared to the baseline (i.e., pre-program) [*t*(22) = 2.52, *p* < 0.05]. Such differences were not found in the relaxation group, as shown in Figure 2 (*p* > 0.05).

The same mixed ANOVAs conducted on RT data for the alerting and orienting networks revealed significant main effects of alerting [*F*(1,35) = 174.45, *p* < 0.05] and orienting [*F*(1,22) = 180.07, *p* < 0.05] conditions. However, the interaction between time and condition was not significant in any group (all *F*s < 1 and *p*s > 0.05). Mean RT and accuracy data of each condition for each group are provided in Table 2.

**TABLE 2:** Mean RT (SD) and accuracy (SD) of each condition for each group.

		**Meditation**				**Control**				**Statistical analyses**	
			
		**No cue**	**Center cue**	**Spatial cue**	**Mean**	**No cue**	**Center cue**	**Spatial cue**	**Mean**	***t***	***p*-value**
Pre	Congruent										
	Accuracy	0.99 (0.04)	0.99 (0.04)	0.99 (0.03)	0.99 (0.04)	0.98 (0.04)	0.99 (0.03)	0.99 (0.02)	0.99 (0.03)	0.743	0.936
	RT (ms)	709.11 (127.70)	649.44 (117.41)	601.99 (107.67)	653.51 (124.24)	770.18 (117.61)	693.46 (99.75)	642.99 (80.57)	702.21 (111.36)	1.314	0.197
	Incongruent										
	Accuracy	0.96 (0.06)	0.96 (0.06)	0.96 (0.07)	0.96 (0.06)	0.96 (0.04)	0.97 (0.05)	0.98 (0.03)	0.97 (0.04)	0.955	0.346
	RT (ms)	810.29 (169.44)	758.50 (151.93)	694.32 (156.41)	754.37 (164.18)	846.69 (107.74)	788.52 (106.79)	733.13 (105.04)	789.45 (114.01)	0.743	0.463
	Mean										
	Accuracy	0.97 (0.05)	0.97 (0.05)	0.97 (0.05)	0.97 (0.05)	0.97 (0.04)	0.98 (0.04)	0.99 (0.02)	0.98 (0.04)	0.641	0.525
	RT (ms)	759.70 (156.92)	703.97 (145.13)	648.15 (140.74)	703.94 (153.63)	808.43 (117.33)	740.99 (112.36)	688.06 (102.69)	745.83 (120.30)	1.005	0.322

Post	Congruent										
	Accuracy	0.98 (0.04)	0.98 (0.03)	0.99 (0.03)	0.98 (0.03)	0.98 (0.04)	0.99 (0.01)	0.99 (0.02)	0.99 (0.03)	0.413	0.682
	RT (ms)	710.93 (117.04)	652.74 (91.74)	607.60 (86.37)	657.09 (106.69)	783.02 (108.99)	727.04 (93.09)	664.47 (96.36)	724.84 (108.91)	2.095	0.043
	Incongruent										
	Accuracy	0.96 (0.05)	0.96 (0.07)	0.97 (0.04)	0.96 (0.05)	0.97 (0.03)	0.97 (0.04)	0.98 (0.03)	0.98 (0.03)	0.955	0.293
	RT (ms)	784.79 (104.43)	723.55 (99.30)	679.24 (111.19)	729.19 (112.33)	881.58 (135.55)	802.58 (120.39)	746.62 (132.70)	810.26 (138.36)	2.160	0.038
	Mean										
	Accuracy	0.97 (0.05)	0.97 (0.05)	0.98 (0.03)	0.97 (0.04)	0.98 (0.04)	0.98 (0.03)	0.99 (0.03)	0.98 (0.03)	0.847	0.403
	RT (ms)	747.86 (115.86)	688.15 (101.07)	643.42 (104.89)	693.14 (114.99)	832.30 (130.71)	764.81 (112.39)	705.55 (121.24)	767.55 (131.00)	2.175	0.036

### Functional Imaging Results

#### Executive Control Network

To follow up on the behavioral findings on improvement in executive attention functions, we conducted factorial analyses on functional imaging data. Specifically, a 2 (Time) × 2 (Group) factorial analysis of the brain contrast of interest (i.e., Incongruent > Congruent) showed significant interactions between group and time in key regions for conflict detection and resolution (Table 3). To further test the separate effects of different types of study programs (meditation vs. relaxation) on the executive control network, we conducted *post hoc* tests using beta coefficient values of the activated voxels of interest (VOIs) from each participant in each group in each condition. First, VOIs were defined by 4-mm-radius spheres around the maxima of the clusters found in the group contrast map: right DLPFC and right ACC. Next, beta values of each VOI were extracted from each contrast of interest (i.e., Incongruent > Congruent) at each time point (i.e., pre- and post-program) for individuals in each group (i.e., meditation and relaxation). The extracted beta values were then entered and analyzed in SPSS 22.0 with 2 (time) × 2 (group) mixed ANOVAs.

**TABLE 3:** Brain areas that showed significant Group × Time interactions for executive functions.

**Contrast**	**Brain regions**	**No. of voxels in cluster**	**Peak coordinates**	***t*-value**	**Corrected *p*-value of cluster**
Group × Time for Conflict resolution	R DLPFC	25	28 32 16	3.3	< 0.001
			52 −32 56	2.84	0.003
	R ACC	25	16 42 20	3.15	< 0.001
	LPrecuneus	32	−10 −60 64	3.15	< 0.001
			−8 −66 58	2.77	0.004

**FIGURE 3:**
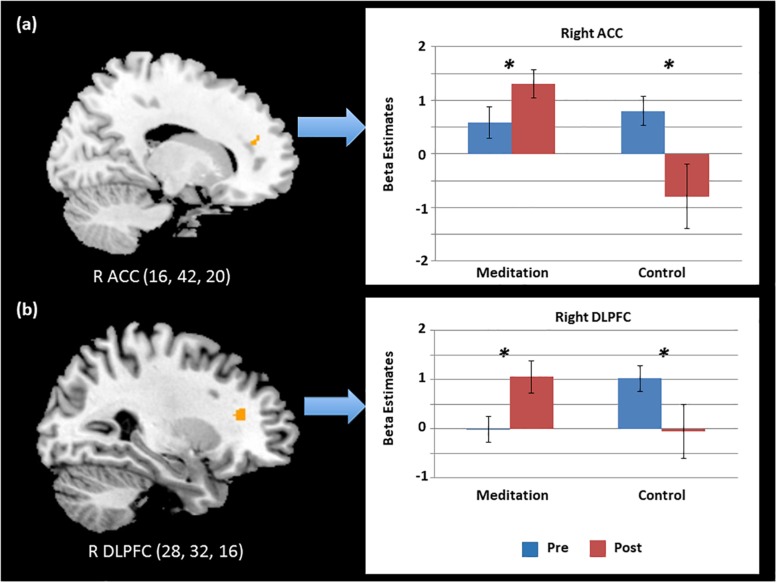
Significant time and group interaction effects in the activation of the **(a)** right anterior cingulate cortex (ACC) and **(b)** right dorsolateral prefrontal cortex (DLPFC) during cognitive conflict. ^∗^*p* < 0.05.

As shown in Figure 3, the results supported our hypothesis that only the intensive and combined meditation in the meditation program improved functions of the executive control network, as the relaxation in a temple did not have such effects. That is, activation in the right DLPFC and right ACC significantly increased after meditation when cognitive conflict existed (i.e., Incongruent > Congruent). Specifically, significant interaction effects between time and group were observed in the right DLPFC [*F*(1,35) = 12.37, *p* < 0.05] and the right ACC [*F*(1,35) = 10.83, *p* < 0.05]. A main effect of group was also significant in the right ACC [*F*(1,35) = 6.39, *p* < 0.05]. *Post hoc t* tests examining the effects of program (Time) in each group confirmed that these effects were driven by significantly increased activation in the meditation group after the program [*t*(22) = 2.07, *p* < 0.05 and *t*(22) = 2.83, *p* < 0.05 in the right ACC and right DLPFC, respectively] and by significantly decreased activation in the relaxation group [*t*(13) = 2.28, *p* < 0.05 and *t*(13) = 2.27, *p* < 0.05 in the right ACC and right DLPFC, respectively]. Further, *post hoc* independent *t* tests testing group effects on brain data from post-programs revealed significant effects of group, confirming that only the intensive meditation program enhanced brain activation in executive control networks [*t*(35) = 3.60, *p* < 0.05 and *t*(35) = 2.04, *p* < 0.05 in the right ACC and right DLPFC, respectively].

**FIGURE 4:**
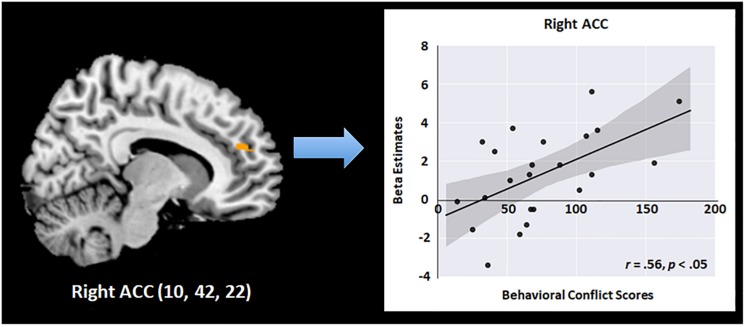
Scatterplots of the relationship between the activation changes in the right ACC and cognitive conflict in behavioral performance in the meditation group (*r* = 0.56, *p* < 0.05).

To further test the effects of meditation on neural functions of executive attention, we conducted a multiple regression analysis using behavioral conflict scores as a factor and brain activity level for executive attention as a dependent variable after the 4 days of the program. The behavioral conflict scores were defined based on RT differences between congruent and incongruent trials in the flanker task performed after the intervention in the meditation group. Neural functions of executive attention were estimated based on brain activity levels in the “Incongruent–Congruent” contrast in fMRI obtained from the meditation group after the program. As a result, the mean conflict score of the meditation group before and after the intervention were 100.86 (SD = 57.90) and 72.10 (SD = 40.64), while those of the relaxation group before and after the intervention were 87.24 (SD = 21.29) and 85.41 (SD = 47.97). The regression analysis results revealed a significant positive relationship between the behavioral conflict scores and neural functions of conflict resolution in the right ACC in the meditation group after the program (Figure 4). Specifically, neural activity in the right ACC regions was significantly predicted by behavioral conflict levels in each individual in the meditation group [*R*^2^ = 0.31, *r* = 0.56, *F*(1,21) = 9.23, *p* < 0.05]. The right ACC was the region that showed significant improvement during the flanker task after the meditation program, as identified in the factorial analysis. This finding thus indicates that, after the meditation program, the more conflict an individual experienced, the greater the neural activity occurred in the right ACC to detect and resolve the attentional conflict. This increase in neural function in turn results in positive effects, as shown by the lower conflict overall in the meditation group compared to the relaxation group after the 4-day program. This relationship between neural functions and behavioral conflict scores was not found in the relaxation group either before or after the program. Further, no relationship was found between behavioral and neural conflict levels in the meditation group before the program, confirming that these effects were induced by the intensive meditative training during the meditation program.

#### Alerting Network

Although no significant interaction effects were found in behavioral performance in the alerting and orienting networks, we conducted the same factorial analyses on neural correlates of alerting and orienting functions as on the executive function to test possible neural changes following the meditation program. Indeed, results from the factorial analysis on the alerting network revealed significant interaction effects on neural networks of alerting functions: bilateral superior temporal gyrus (STG) and left insula (Table 4). Specifically, only people in the meditation group showed significant improvement in such neural correlates of the alerting network after the meditation program, whereas participants in the relaxation group did not.

**TABLE 4:** Brain areas that showed significant Group × Time interactions for alerting functions.

**Contrast**	**Brain regions**	**No. of voxels in cluster**	**Peak coordinates**	***t*-value**	**Corrected *p*-value of cluster**
Group × Time for the	R medial frontal gyrus	318	8 −16 64	4.91	< 0.001
Alerting network	R STG	157	48 −34 14	4.32	< 0.001
(Center Cue > No Cue)			56 −32 12	3.77	< 0.001
	LSTG	230	−56 −28 0	3.97	< 0.001
			−48 −18 −8	3.58	0.003
	L Insula	32	−10 −60 64	3.76	< 0.001
	LClaustrum		−34 −22 10	3.16	0.001
	R STG	25	68 −18 0	3.45	< 0.001
	RSTG	49	52 −20 −4	3.25	0.001

#### Orienting Network

As in the alerting network, the results of the factorial analysis on the orienting network also showed significant interaction effects of group and time on neural networks of attentional orienting: right DLPFC, right superior frontal gyrus (SFG) and frontal eye field (FEF), bilateral inferior frontal gyrus (IFG), and right ACC (Table 5). That is, participants in the meditation group showed significantly increased activation in the frontal attentional orienting networks after the meditation, whereas participants in the relaxation group did not show the same increase. Together with findings from the alerting networks, these results indicate neural changes after the 4-day intensive meditation retreat, although behavioral measures for those two attention networks might not be sufficiently sensitive to detect such effects.

**TABLE 5:** Brain areas that showed significant Group × Time interactions for orienting functions.

**Contrast**	**Brain regions**	**No. of voxels in cluster**	**Peak coordinates**	***t*-value**	**Corrected *p*-value of cluster**
Group × Time for the	R DLPFC	55	54 36 18	4.63	< 0.001
Orienting network	L ACC	28	−14 20 30	3.75	< 0.001
(Spatial Cue > Central Cue)			−12 28 26	2.88	0.003
	L Middle frontal gyrus	281	−28 62 12	3.73	< 0.001
			−32 54 14	3.47	< 0.001
	LCingulate Gyrus	51	−16 2 46	3.63	< 0.001
	LIFG	24	−52 40 4	3.49	< 0.001
			−54 34 10	2.91	0.002
	R IFG	39	28 28 −2	3.48	< 0.001
	LMedial frontal gyrus	82	−14 54 −4	3.41	0.001
			−14 36 −12	3.18	0.001
	R SFG/FEF	67	22 58 30	3.4	0.001
			16 62 26	3.27	0.001
	R ACC	26	10 30 10	3.34	0.001
	R ACC	21	18 28 20	3.27	0.001
	R ACC	29	14 46 −2	3.07	0.002

## Discussion

In the present study, we used an ANT task to investigate the behavioral and neural effects of a short-term meditation retreat on attentional function. Our results provided novel findings that executive attention functions were improved after meditation at both behavioral and neural levels. In addition, participants showed enhanced neural function in the brain areas related to the alerting and attentional orienting. Finally, increased neural functions associated with conflict resolution in the right ACC revealed a significant positive relationship with the behavioral conflict scores in the meditation program. Based on such results, we suggest that attentional functions, especially executive attention, were improved after the short-term meditation retreat.

### Changes in the Executive Control Network Both at the Behavioral and Neural Levels

The present study demonstrates changes in the executive control network both at the behavioral and neural levels after a short-term meditation retreat. At the behavioral level, previous studies have consistently reported effects of short-term meditation on the attentional network. Studies identified improvements in conflict scores on the ANT after a few days of 20- to 60-min meditation training (van Veen and Carter, 2002; Ainsworth et al., 2013). Our behavioral results also support previous findings. However, none of the previous studies examined the effect of meditation on neural correlates of executive attention via task fMRI. In addition to the changes in behavioral performance, here we observed that neural activities in the ACC and DLPFC were increased during the ANT after meditation. As previously reported, in a situation that requires control of cognitive conflict, the ACC monitors and detects the occurrence of conflict and sends signals to other brain regions, such as the DLPFC, to implement cognitive control (Mansouri et al., 2009). The changes in behavioral data are known to be highly associated with changes in the neural level. In the previous studies, decreased brain activation of the attention-related regions is often accompanied with poor performances during the attention task, especially in the individuals with attentional deficit (Adler et al., 2005; Rubia, 2009). Moreover, brain activation was boosted after psychological treatment along with the enhancement of cognitive task performance. In our results, it is likely that the increased activations of the ACC facilitate the resolution of cognitive conflict during the ANT. That is, the strong correlation between the changes in ACC activation and behavioral conflict scores supports the idea that these neural changes in the meditation group are associated with improvement of the executive control network.

Previous studies have suggested that the change in brain activation levels depends on the stages of meditation practice – early, intermediate, and advanced – and showed a quadratic relationship (Lazar et al., 2000; Tang et al., 2015). Each stage requires a different amount of effort to exercise self-regulation and induces different levels of activation in the brain. In the early stage, the participant exerts effortful control to stay engaged in meditation, with increased activation in the ACC and lateral PFC. Next, the intermediate stage requires somewhat less effort to reduce mind wandering, and little or no effort is needed in the advanced stage. Therefore, those with higher levels of meditation expertise show less activation in the brain areas that showed increased activation in the early stage. Because all of our participants were novices, having practiced meditation for only 4 days, the increased ACC activation observed after the meditation also supports this model.

### Changes in the Alerting and Orienting Network at the Neural Level

Surprisingly, the alerting and orienting networks also showed significantly increased brain activation, despite the absence of significant improvement in the behavioral data. Previously, such changes in the alerting and orienting networks had been reported only in long-term meditation studies (van Veen and Carter, 2002; Jha et al., 2007). As a possible explanation, we considered that those networks may be affected by short-term meditation but change more slowly than does the executive control network. Based on the previous fMRI studies, the brain regions involved in the alerting networks are the insula and temporal regions, and those involved in the orienting networks are the parietal and superior frontal regions (Fan et al., 2005). Compared to other brain areas, the PFC, which is mainly involved in the executive control network, is known to display remarkable functional plasticity throughout life (McEwen and Morrison, 2013). Such plasticity may facilitate improvements of the executive control network within a few days, while other attentional networks might require more time to show distinct changes. Further studies are necessary to clarify whether changes in the alerting and orienting networks are markedly affected by functional plasticity of the brain regions.

### Minimization of Non-meditative Factors

Since a decade ago, Templestay, used as an invention in the current study, has been a wide-spreading program in Korean Buddhist temples. The program allows individuals to experience the daily routines of Buddhist monks, which is lifestyle with mindful meditation. The effect of this program had been proved in previous studies, but the possible effects from the changes on environment and diet were hard to be excluded from the effect of meditation. To elucidate the meditation effect of Templestay, we included a relaxation program, which shared the same environment and diet with Templestay program, but without any meditative factors. Our result suggested that increased executive control network in the meditation group is not induced by diet or vacation effect, but solely from the meditation practice.

### Limitation

Despite its novelty, the present study has several limitations. First, about 20% of fMRI data were removed from the analyses due to excessive movement. As mentioned in the procedure, our study was a within-subject design in which each participant participated both pre- and post-fMRI task (i.e., before and after Templestay). Thus, we excluded fMRI data if participants moved excessively at least one of the two fMRI sessions (i.e., pre- or post-), which resulted to remove approximately 20% of the samples in our study. Future research with more samples and having practice mock scans before actual scans would help to increase power to detect changes in attention networks after meditation. Another limitation is a lack of a relaxation group that underwent a rest period in a general environmental setting, rather than in the Buddhist temple. Including that type of control might be able to provide an additional comparison to the intervention groups that stayed in the temple and allow examination of the effects of staying at the temple and changing one’s diet. However, due to the difficulty of controlling the variation in participants’ resting environments and the neural effects of that variation, we chose a relaxation group that rested freely in the same temple environment. We believe that our study design had strong benefits for ruling out the confounding effects of food intake and surroundings during the meditation program on cognitive functions. For further studies, randomized controlled trials with three groups (meditation, residential-controlled active control, and pure control groups) were suggested to clarify the effects of individual factors of meditation on attentional functions. The second limitation is a lack of a personality trait variable. Several studies suggested a possible relationship of meditation practices with personality of individuals (Shapiro et al., 2011; Nyklicek et al., 2013; Tang et al., 2017). These studies suggested that the differences in personality may associated with how people respond to and benefit from meditation practice. However, more empirical studies are encouraged to elucidate the interaction between personality traits and meditation. Last, the current study had not been pre-registered.

## Conclusion

In conclusion, we present novel evidence that short-term meditation changed brain functions of attention and thereby improved the attentional networks. Given the growing demands for interventions based on meditation, our findings provide insight into the neural changes in the attentional network induced by short-term meditation in both healthy people and individuals at risk of attention deficits. Training to improve attention has the potential to be beneficial in various fields, as attention networks are essential to higher-order cognitive operations.

## Data Availability Statement

The datasets generated for this study are available on request to the corresponding author.

## Ethics Statement

All participants provided written informed consent for participation in accordance with the Declaration of Helsinki, and the protocol was approved by the Institutional Review Board of Seoul National University Hospital Committee.

## Author Contributions

SK and S-YK: acquisition of data; analysis; manuscript interpretation, making figures, revision and final approval of the manuscript; agreement to be accountable for all aspects of the work. TL: interpretation, critical comments on the manuscript, revision and final approval of the manuscript; agreement to be accountable for all aspects of the work. DB, KC, and W-JH: acquisition of data; revision and final approval of the manuscript; agreement to be accountable for all aspects of the work. K-OL, S-YK, H-YP, and JK: acquiring fund, conception and design of the work; interpretation, critical comments on the manuscript, revision and final approval of the manuscript; agreement to be accountable for all aspects of the work.

## Conflict of Interest

The authors declare that the research was conducted in the absence of any commercial or financial relationships that could be construed as a potential conflict of interest.
