# Supplementary figures and images for: Enhanced Attentional Network by Short-Term Intensive Meditation
Source: Front Psychol. 2020 Feb 7;10:3073. doi: 10.3389/fpsyg.2019.03073 (PMC7019009; doi:10.3389/fpsyg.2019.03073)

## Supplementary Material

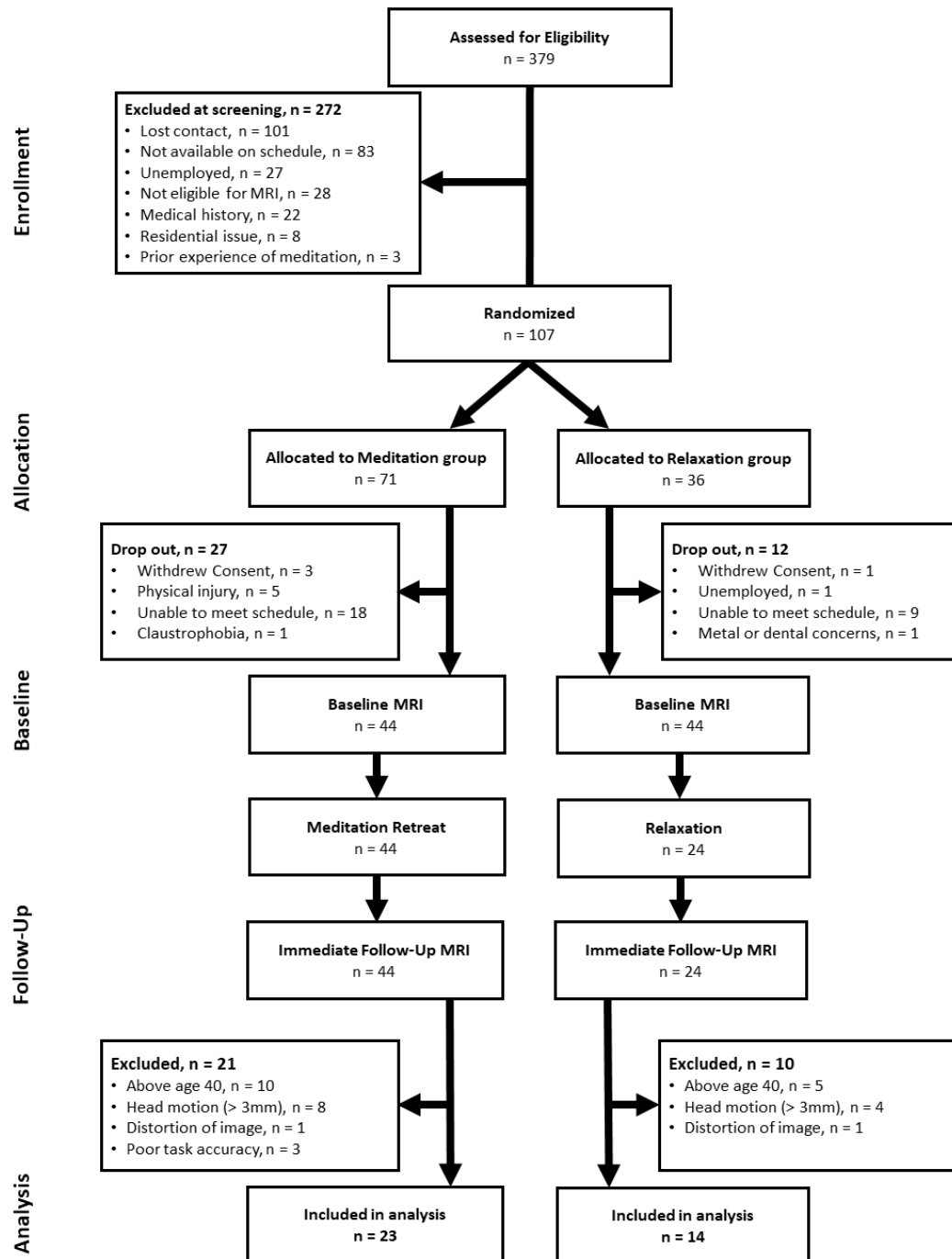

Supplementary Figure 1. Flowchart of the study

Supplement: Supplementary file 1 [file Image_1.pdf]
